# Live Imaging of Bioluminescent *Leptospira interrogans* in Mice Reveals Renal Colonization as a Stealth Escape from the Blood Defenses and Antibiotics

**DOI:** 10.1371/journal.pntd.0003359

**Published:** 2014-12-04

**Authors:** Gwenn Ratet, Frédéric J. Veyrier, Martine Fanton d'Andon, Xavier Kammerscheit, Marie-Anne Nicola, Mathieu Picardeau, Ivo G. Boneca, Catherine Werts

**Affiliations:** 1 Institut Pasteur, Unité Biologie et Génétique des parois bactériennes, Paris, France; 2 INSERM, équipe Avenir, Paris, France; 3 Institut Pasteur, Unité des infections bactériennes invasives, Paris, France; 4 ENS Cachan, département de Biologie, Paris, France; 5 Institut Pasteur, Plate forme d'imagerie dynamique, Paris, France; 6 Institut Pasteur, Unité Biologie des Spirochètes, Paris, France; Medical College of Wisconsin, United States of America

## Abstract

*Leptospira (L.) interrogans* are bacteria responsible for a worldwide reemerging zoonosis. Some animals asymptomatically carry *L. interrogans* in their kidneys and excrete bacteria in their urine, which contaminates the environment. Humans are infected through skin contact with leptospires and develop mild to severe leptospirosis. Previous attempts to construct fluorescent or bioluminescent leptospires, which would permit *in vivo* visualization and investigation of host defense mechanisms during infection, have been unsuccessful. Using a firefly luciferase cassette and random transposition tools, we constructed bioluminescent chromosomal transformants in saprophytic and pathogenic leptospires. The kinetics of leptospiral dissemination in mice, after intraperitoneal inoculation with a pathogenic transformant, was tracked by bioluminescence using live imaging. For infective doses of 10^6^ to 10^7^ bacteria, we observed dissemination and exponential growth of leptospires in the blood, followed by apparent clearance of bacteria. However, with 2×10^8^ bacteria, the septicemia led to the death of mice within 3 days post-infection. In surviving mice, one week after infection, pathogenic leptospires reemerged only in the kidneys, where they multiplied and reached a steady state, leading to a sustained chronic renal infection. These experiments reveal that a fraction of the leptospiral population escapes the potent blood defense, and colonizes a defined number of niches in the kidneys, proportional to the infective dose. Antibiotic treatments failed to eradicate leptospires that colonized the kidneys, although they were effective against *L. interrogans* if administered before or early after infection. To conclude, mice infected with bioluminescent *L. interrogans* proved to be a novel model to study both acute and chronic leptospirosis, and revealed that, in the kidneys, leptospires are protected from antibiotics. These bioluminescent leptospires represent a powerful new tool to challenge mice treated with drugs or vaccines, and test the survival, dissemination, and transmission of leptospires between environment and hosts.

## Author Summary

Leptospirosis is a worldwide neglected disease caused by the pathogenic bacterium named *Leptospira interrogans*. Some rodents, such as rats, do not get sick from leptospirosis and constitute a reservoir. They carry leptospires in their kidneys and excrete the bacteria in the environment. *L. interrogans* are mobile and penetrate their hosts through abraded skin or mucosa. Infected humans may develop mild to severe leptospirosis, potentially leading to death. Leptospires are difficult to cultivate and to genetically manipulate, impairing the study of leptospirosis. Here, we constructed bioluminescent leptospires, and monitored infection in live mice by tracking bioluminescence. In the first days after infection, a rapid dissemination and growth of bacteria was observed in the blood circulation, followed around one week after the infection by their apparent disappearance. However, the leptospires reemerged and multiplied in the kidneys, to reach sustained levels three weeks after infection. The use of antibiotics showed that antibiotic-susceptible *L. interrogans* are very difficult to eradicate once they are settled in the kidneys. Mice infected with bioluminescent leptospires represent a pertinent model to study leptospirosis. These bioluminescent leptospires are novel tools that will be useful to test the efficacy of treatments or vaccines against leptospirosis.

## Introduction

Leptospirosis is a worldwide zoonosis transmitted by chronically infected animals, through excretion of bacteria in their urine, contaminating the soil and water. Pathogenic *Leptospira interrogans (L. interrogans)* are spiral motile bacteria that enter a diverse range of hosts through broken skin and mucosa. Infected humans develop either a flu-like, usually mild illness, or severe disease with renal, liver and heart failure, and eventually pulmonary hemorrhages that may be fatal. Leptospirosis is a reemerging and under-diagnosed neglected zoonotic disease, that may increase worldwide because of global warming and uncontrolled urbanization [1]. In East Asia, leptospirosis is a serious health issue affecting paddy field workers, and people living in regions often flooded after typhoons. Frequent outbreaks of leptospirosis occur in developing countries, where a large part of the population lives in slums with poor urban sanitation, in close contact with infected rats. In Europe, leptospirosis affects people in contact with animals, or taking part in outdoors activities in water environments [2]–[4]. The pathophysiology of leptospirosis remains poorly understood. Leptospirosis has been studied in animal models by immunohistochemistry to assess pulmonary, hepatic and renal lesions [5], [6]. Different techniques, such as direct isolation and subsequent culture of leptospires from organs and body fluids, electron microscopy and more recently, quantitative real time polymerase chain reaction (q-PCR) of leptospiral DNA, have been very useful to better understand the course and burden of leptospires in their hosts [7]–[9]. Nowadays, *in vivo* imaging of small live animals is easily performed, allowing non-invasive longitudinal monitoring of disease progression using luminescent or fluorescently labeled microbes [10]. One such study has recently been performed with *L. interrogans* in transparent Zebrafish embryos, demonstrating fast uptake of leptospires by phagocytic cells [11]. However to our knowledge, no dynamic study of the *in vivo* dissemination of leptospires has been undertaken in mammals. Two teams have succeeded in constructing genetically modified leptospires species expressing either fluorescent GFP or mRFP proteins [12] or luciferase from a luxCDABE cassette from *Photorhabdus luminescens* [13]. These labeled strains proved to be useful tools to easily enumerate the bacteria or for use in *in vitro* assays. However, these constructs did not allow the monitoring of infection with *L. interrogans* in cells or in animals. In contrast, a recent study showed the feasibility of using the firefly luciferase gene under the control of a strong promoter to obtain luminescent *Borrelia* species and track the bacteria *in vivo* in mice [14]. Therefore, we chose to construct bioluminescent leptospires, introducing a cassette expressing the firefly luciferase gene under the control of a strong leptospiral promoter, by transposon insertion in the chromosome of leptospires [10]. Since the reaction catalyzed by the firefly luciferase in the presence of its substrate, D-luciferin, requires ATP to emit photons, only metabolically active and therefore live bacteria expressing the firefly luciferase are bioluminescent in the presence of D-luciferin.

Until recently, mice have not been regarded as a good model for leptospirosis, as they are considered resistant to acute leptospirosis, typically surviving the infection [8]. However, we and another group have demonstrated that *L. interrogans*-infected C57BL/6J mice develop mild fibrosis, associated with chronic renal carriage of leptospires [15], [16]. Hence, in this work we used bioluminescent strains of *L. interrogans* to visualize and characterize the course of leptospirosis in mice, and study the efficacy of antibiotic treatments, administered at different phases of the infection.

## Materials and Methods

### Bacterial strains and culture conditions

*L. biflexa* sevorar Patoc strain Patoc (Paris) and *L. interrogans* serovar Manilae strain L495 were used in this study as described earlier [17]. Bacteria were grown in Ellinghausen-McCullough-Johnson-Harris (EMJH) medium (Bio-Rad) at 28°C without agitation. EMJH agar plates were obtained by solidification of EMJH medium with 1.2% noble agar (Difco). Leptospires were counted using a Petroff-Hauser chamber. Antibiotics were used *in vitro* at the following concentrations: 100 µg/mL ampicillin (Amp, MP Biomedical); 25 µg/ml kanamycin (Km, Sigma-Aldrich); 50 µg/ml spectinomycin (Spc, Sigma-Aldrich). Thymidine (dT, Sigma-Aldrich) and diaminopimelate (DAP, Sigma-Aldrich) were added when necessary at the final concentration of 0.3 mM.

### Construction of pCj::FlgB plasmid

The firefly luciferase gene was amplified from the vector p5L3::Km [18] using the LucNcoIF and LucPstIR primers (Table 1) and ligated into pBAD28 (Life Technologies) using NcoI and PstI restriction enzymes to generate pBAD::Luc. A strong leptospiral promoter *flgB*p, encompassing the upstream region of the flagellar basal-body rod protein, has been amplified from *L. interrogans* Fiocruz L1-130 DNA (location chromosome 1 342199-341959) using specific primers (Table 1). The promoter was independently subcloned into pBAD::Luc using NcoI and NheI, resulting in the pFlgB::Luc plasmid expressing the firefly luciferase gene under the control of the leptospiral promoter. The luciferase cassette was recovered from the pFlgB::Luc plasmid using the NheI/PvuI enzymes. Plasmid pCj::FlgB was generated by cloning the blunt-ended luciferase cassette into the blunt-ended PacI restriction site of the suicide conjugative plasmid pCjTKS2 [19].

**Table 1:** Primer list for cloning.

*Primer name*	*Sequence (5′* to *3′)*
LucNcoIF	GTA**CCATGG**AAGACGCCAAAAACATAAAG
LucPstIR	ATACAG**CTGCAG**CCTACAATTTGGACTTTC
pFlgBF	ACT**GCTAGC**AATAGAATTCATAATTGGAAT
pFlgBR	CAC**CCATGG**TTTCTCCCCCTTCTCAAAA

Boldface indicates restriction sites.

### Transposon mutagenesis and conjugation between *E. coli* and leptospires

Random transposition of the luciferase cassette was conducted as previously described [20]. Briefly, pCj::pFlgB plasmid was transformed into *E. coli* β2163 (DAP^−^, [21]) by heat shock at 42°C for 45 s and transformants were selected on Luria Bertani medium plates containing kanamycin and DAP. *L. interrogans* and *L. biflexa* strains were grown to mid exponential phase (2×10^7^ bacteria/mL) in liquid EMJH. Conjugation between *E. coli* β2163 carrying pCj::pFlgB and leptospiral strains was done as previously described [22] and the transformants selected on EMJH plates containing kanamycin. The unknown insertion junction fragment of each transformant was determined by semi-random PCR and sequenced as described [22].

### Infection experiments with leptospires

Female mice (7- to 10-wk old) were used in this study. Female Albino C57BL/6J mice were purchased from Charles Rivers (Chatillon-sur-Chalaronne, L'Arbresle Cedex, France). Balb/c and C57BL/6J mice were from Janvier (Le Genest, France).

Infections with leptospiral strains and bioluminescent mutants were conducted as described earlier [17]. Just before infection, bacteria in late exponential phase (around 10^8^ leptospires *per* ml) were centrifuged at room temperature for 25 min at 3250 ×g, resuspended in endotoxin-free PBS, and counted using a Petroff-Hauser chamber. 200 µl of leptospires in PBS were injected via the intraperitoneal route into mice.

### Bioluminescence imaging

D-luciferin potassium salt (Caliper Life Sciences), the substrate of Firefly luciferase, was dissolved in PBS at a concentration of 30 mg/ml. 10 minutes before imaging, 100 µl (3 mg) of D-luciferin was given to mice by intraperitoneal injection. Mice were then anesthetized using a constant flow of 2.5% isoflurane mixed with oxygen and air as recommended by the manufacturer, using an XGI-8 anesthesia induction chamber (Xenogen Corp.). Mice were maintained in the anesthesia chamber for at least 5 min to allow adequate dissemination of the injected substrate. Bacterial infection images were acquired using an IVIS Spectrum system (Xenogen Corp., Alameda, CA) according to instructions from the manufacturer. Analysis and acquisition were performed using Living Image 3.1 software (Xenogen Corp.). Images were acquired using 5 min of integration time with a binning of 8 and with the emission filter in the “open” mode. All other parameters were held constant. Quantification was performed using a region of interest defined manually (whole mouse or kidneys) and the results were expressed as photons (P) per second (s) per cm^2^ per steradian (SR). Non-infected control mice and infected mice were separately imaged in order to prevent hypothetical contamination. At different time points after infection, 50 µl of blood was collected at the retro-mandibular sinus, added to 20 µl of 100 mM EDTA and imaged immediately after addition of 10 µl of 3 mg/ml D-luciferin in PBS, to detect viable bacteria, or 10 µl of a solution of 3 mg/ml D-luciferin in PBS complemented with 1 mM ATP, to detect both live and dying bacteria. Same procedure was used to image bioluminescent *L. interrogans* in EMJH medium or in urine, collected holding the mouse above a sterile Petri dish. For *ex vivo* analyses, mice were euthanized, and the kidneys and organs removed, cut and imaged for 5 min after immersion in D-luciferin solution (3 mg/ml in PBS), then stored at −80°C for subsequent detection of leptospires by q-PCR.

### Antibiotic treatments

Penicillin G (Sigma), ciprofloxacin (Fluka/Sigma-Aldrich), azithromycin (Azi, RTC Sigma) and doxycycline (Sigma-Aldrich) were used at the equivalent human dose of 150 000 units/kg/day, 50 mg/kg/day, 25 mg/kg/day, and 3,3 mg/kg/week, respectively. Antibiotics were diluted in endotoxin-free PBS (Biowhittaker). To solubilize ciprofloxacin and azithromycin, the pH was dropped to 6 with 1N HCL. Antibiotic solutions were sterilized by filtration (0.45 µm) and a volume of 100 µl was administered daily to ∼20 g albino C57BL/6J mice via the intraperitoneal (IP) route for 5 consecutive days, either beginning one day post infection (dpi), or 3 dpi, or for 7 consecutive days during the chronic phase of infection. For prophylaxis experiments, a single IP injection of azithromycin or doxycycline was carried out 2 days prior to the infection.

### Leptospiral loads

The leptospiral burden in urine, blood and organs was determined by quantitative real-time PCR (q-PCR), as described [15]. The Maxwell 16 automat was used to extract total DNA from a drop of urine (5 to 100 µl) or from 50 µl of blood, using the cell LEV DNA, or blood DNA purification kits (Promega), respectively. Total DNA from organs was extracted with the DNAeasy tissue Qiagen kit after mechanical disruption with metallic beads. Primers and probe designed in the *lpxA* gene of *L. interrogans* strain Fiocruz [23] were used to specifically detect pathogenic *Leptospira spp* [15]. *L. biflexa* was detected using the classical RNA 16S gene q-PCR, as described [8]. q-PCR reactions were run on a Step one Plus real-time PCR apparatus using the absolute quantification program (Applied Biosystems), with the following conditions for FAM TAMRA probes: 50°C for 2 min, 95°C for 10 min, followed by 40 cycles with denaturation at 95°C for 15 s and annealing temperature 60°C for 1 min, according to the manufacturer's instructions. Results were expressed as the number of leptospires per 100 µl of urine, 50 µl of blood, or *per* 200 ng of total DNA extracted from organs.

### Ethics statement

All protocols were reviewed by the Institut Pasteur (Paris, France), the competent authority, for compliance with the French and European regulations on Animal Welfare and with Public Health Service recommendations. This project has been reviewed and approved (# 2013-0034) by the Institut Pasteur ethic committee (CETEA #89).

### Statistical analysis

Statistical analysis was performed using Graph Pad Prism software. The unpaired *t* test, (two-tailed P values) was used to compare two groups at the same time point. Values are expressed as mean ± standard error of the mean (SEM). A *p* value <0.05 was considered significant. *p* values: ^*^ *p*<0.05, ^**^ *p*<0.01, ^***^ *p*<0.001.

## Results

### Production of bioluminescent transformants of *L. interrogans and L. biflexa*

Bioluminescent transformants of *L. interrogans* and *L. biflexa* were successfully obtained after chromosomal insertion of the firefly luciferase cassette by random transposition, as previously described [20]. All kanamycin resistant individual colonies of leptospires selected on agar plates after conjugation were checked for bioluminescence. The chromosomal insertion site of the transposed luciferase cassette was determined after amplification of the flanking regions by semi nested PCR and sequencing of the PCR products (Table 2), as previously described [22].

**Table 2:** List of bioluminescent leptospires transformants obtained in this study.

Strain	Mutant name	Location of insertion^a^	Predicted function of mutated gene or description
*L. interrogans* Manilae	*MFlum1*	*LMANv1_3260005 LMANv1_3260004*	*Conserved protein – Putative lipoprotein*
	MFlum2	LMANv1_3260005	Conserved exported protein of unknown function
	MFlum3	LMANv1_2140001	Conserved protein of unknown function
	MFlum4	LMANv1_4300006	O-acetylhomoserine sulfhydrylase
	MFlum5	LMANv1_6000004	Protein of unknown function
	*MFlum9*	*LMANv1_7240005 LMANv1_7260001*	*Thiamine-monophosphate kinase – 30S ribosomal protein*
	MFlum10	LMANv1_3330001	Cytoplasmic membrane protein
	MFlum16	LMANv1_130012	Histidine kinase sensor protein
	MFlum17	LMANv1_1720002	Microbial collagenase
	MFlum18	LMANv1_8780007	Two-component sensor histidine kinase VicK
	*MFlum28*	*LMANv1_1890005 LMANv1_1890004*	*Conserved protein – 6-pyruvoyl tetrahydrobiopterin synthase*
*L. biflexa* Patoc	PFlum1	LEPBIa2303	Thiamine biosynthesis protein ThiC
	PFlum2	LEPBIa2357	Hypothetical protein
	PFlum3	LEPBIa0902	Putative Mn2+ and Fe2+ transporter (NRAMP-family transporter NCBI = ABZ93401.1)
	PFlum5	LEPBIb0087	Putative acriflavine resistance protein D; putative transmembrane protein
	*PFlum7*	*LEPBIa0480 LEPBIa0481*	*Mannose-1-phosphate guanylyltransferase (GMP) – Conserved hypothetical protein*
	PFlum8	LEPBIa2172	Hypothetical protein
	PFlum10	LEPBIa2309	Conserved hypothetical protein
	PFlum11	LEPBIa3340	Conserved hypothetical protein
	PFlum12	LEPBIa2691	Citrate synthase 1
	PFlum14	LEPBIa0846	DNA-binding ATP-dependent protease

a as annotated in MicroScope (http://www.genoscope.cns.fr/agc/microscope/home/index.php).

italics: insertion in an intergenic region.

A total of 63 bioluminescent transformants of *L. interrogans* serovar Manilae were obtained with the *flgB*p promoter upstream of the luciferase cassette and therefore named “MFlum”. The chromosomal insertion was localized for 11 transformants, and found to be intergenic in 3 clones (MFlum1/9/28). Fourteen transformants were obtained in the *L. biflexa* serovar Patoc named “PFlum”. The insertion site was localized for 13 transformants, and found to be intergenic for one clone (PFlum7) (Table 2).

### *In vitro* characterization of MFlum1

**Figure 1:**
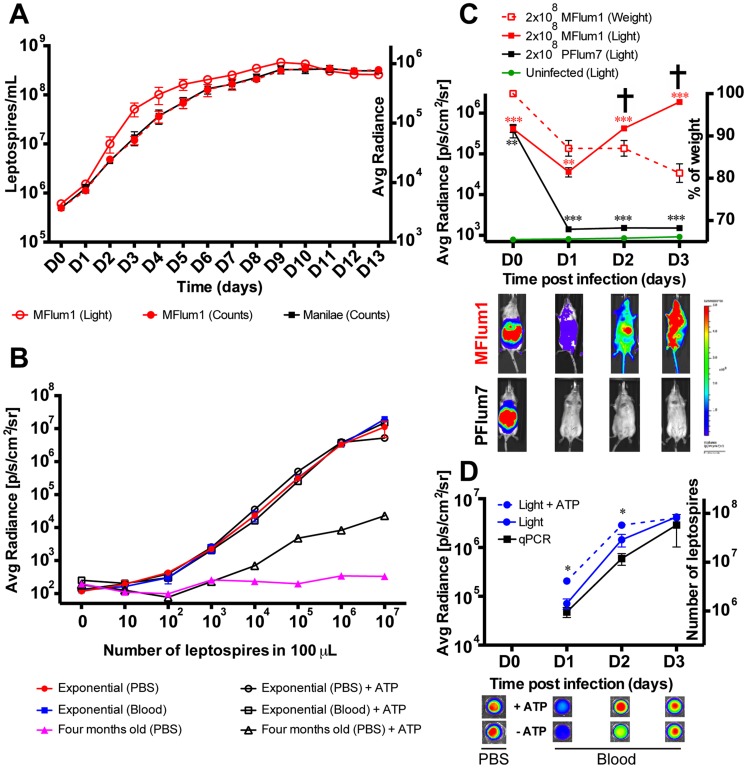
Characterization of bioluminescent *Leptospira* transformants. (A) Growth curves of *L. interrogans* Manilae wild-type and MFlum1 strains in EMJH medium at 28°C (Left Y axis) and corresponding bioluminescence of MFlum1 (Right Y axis). (B) Comparison of bioluminescence according to known numbers of MFlum1, grown to mid-log phase or to old stationary phase (four months). Measures were done with the IVIS Spectrum machine after addition of D-luciferin, in the presence or absence of ATP. Panels A and B are representative of 6 and 2 experiments, respectively. (C) Live imaging tracking over time of 2×10^8^ MFlum1 or bioluminescent *L. biflexa* Patoc PFlum7 injected intra-peritoneally (IP) into albino C57BL/6J mice (Left Y axis) and corresponding weight losses (Right Y axis) of MFlum1 infected mice. All bioluminescence analyses were carried out after the IP administration of D-luciferin. Data are expressed as the mean ± SEM of average radiance of light measured in photons/second/cm^2^ in mice and imaged in the ventral view. This panel represents 3 experiments with a total of n = 12 mice infected with MFlum1, n = 4 mice infected with PFlum7, and n = 8 naïve mice. *p* values (*p<0.05, **p<0.01, ***p<0.001) between infected and uninfected group. The cross indicates that the mice died or were sacrificed because of acute leptospirosis. Below are shown images of the tracking of one mouse photographed at different time post infection. (D) Live imaging tracking over time of 50 µl of blood collected from the 2×10^8^ MFlum1 infected mice at different time points from the experiment shown in panel 1C (Left Y axis) and corresponding number of leptospires measured by q-PCR (Right Y axis). Data are expressed as the mean ± SEM of average radiance of light measured in photons/second/cm^2^ in 50 µL of blood. *p* values (*p<0.05), between groups. This panel represents 2 experiments with a total of n = 6 mice. Below are shown the corresponding images at different dpi in the presence or the absence of ATP. Below D0 are shown images of the MFLum1 in PBS imaged just before IP injection.

Our aim was to construct bioluminescent strains of *L. interrogans* to track the bacteria *in vivo*. Therefore, we choose to characterize the intergenic transformant MFlum1 of *L. interrogans* serovar Manilae for which the transposon insertion should not affect the expression of surrounding genes. No difference of *in vitro* growth in liquid EMJH was found between MFlum1 and the parental Manilae strain (Fig. 1A), with respective generation times of 18.83±0.98 h and 18.01±1.04 h (n = 10 independent experiments, *p*: 0,73), suggesting that the insertion of the cassette was not deleterious to the bacteria, grown *in vitro*. The light emitted by MFlum1 in the presence of the D-luciferin substrate, followed the growth curve (Fig. 1A). The emitted light was strictly proportional to the number of enumerated leptospires harvested in exponential phase, with no saturation up to 10^7^ bacteria in 100 µl of PBS (Fig. 1B). The limit of detection was 10^2^ MFlum1 in 100 µl of PBS (Fig. 1B). In comparison, bacteria from a four-month old culture, were only barely bioluminescent (Fig. 1B). Because they retained their shape under the dark field microscope, we considered the possibility that these nearly metabolically inactive bacteria could still emit light if ATP was provided in addition to the substrate. As ATP is negatively charged, it does not penetrate viable bacteria, however it could reveal the activity of luciferase released into the buffer, or still associated with senescent bacteria with altered membranes. To test this hypothesis, we repeated the dose-response experiments in the presence of D-luciferin and ATP. Indeed, the addition of ATP partially restored the bioluminescence of the bacteria harvested from the old culture, demonstrating that some luciferase protein was still active, and suggesting the recent viability of the bacteria that released the luciferase (Fig. 1B). By contrast, the addition of ATP did not change the bioluminescence of MFlum1 harvested at the exponential phase of growth, suggesting that all the bacteria were metabolically active in exponential phase (Fig. 1B). As we aimed to study septicemia, we verified that blood did not interfere with the emitted light. Bioluminescence of MFlum1, harvested from an exponential culture, was equivalent when diluted in blood or PBS (Fig. 1B). Together these experiments suggest the feasibility of detecting in blood both live and dying MFlum1 still expressing active luciferase, by imaging in presence of D-luciferin and ATP.

### Acute infection of mice with virulent bioluminescent *L. interrogans*

Albino C57BL/6J mice, allowing live imaging because of their white fur, were infected with MFlum1 or as a control, with the bioluminescent saprophytic *L. biflexa* PFlum7. Mice were infected through the intraperitoneal (IP) route with 2×10^8^ bacteria, the infective dose used in our previous studies with *L. interrogans* strain Fiocruz [8], [17]. Dynamic bioluminescence imaging showed that 30 min after infection, both MFlum1 and PFlum7 bacteria were present in the peritoneal cavity (Fig. 1C, lower panel). One day post infection (dpi), the bioluminescence of the PFlum7 strain decreased sharply to a weak but significantly higher level than the background of uninfected mice (Fig. 1C). The PFlum7 signal decreased further to background levels 14 dpi, without recurrence of the bacteria one month post infection (Fig. S1 in S1 Text), showing that, as expected, the saprophytic strain was not infectious. The bioluminescence of *L. interrogans* MFlum1 decreased until 1 dpi, corresponding to the dissemination of bacteria in the whole circulation (Fig. 1C, lower panel), then exponentially increased. However, mice infected with MFlum1 died or were sacrificed 2 or 3 dpi, because they suffered from acute leptospirosis, as mirrored by their weight loss, between 10 to 20% of their initial weight within the first 3 dpi (Fig. 1C, left scale). To exclude the possibility that the MFlum1 transformant gained virulence due to the cassette insertion, we compared the survival of C57BL/6J mice infected with the parental Manilae and MFlum1 strain. MFlum1 was virulent and killed all mice at the dose of 2×10^8^ bacteria, although attenuated compared to the Manilae parental strain since 3 out of 4 mice survived the infection at the dose of 2×10^7^ bacteria (Table 3). These data suggest that the biological cost of luciferase cassette expression has only minor effects on the virulence of the *L. interrogans* MFlum1 transformant.

**Table 3:** Survival test of C57BL/6J mice to the infection with *L. interrogans* Manilae and its mutant MFlum1.

	Infected with WT strain		Infected with MFlum1	
	2×10^7^	2×10^8^	2×10^7^	2×10^8^
**4 days post infection**	4/4 dead	4/4 dead	0/4 dead	4/4 dead
**10 days post infection**	–	–	1/4 dead	–

To ascertain whether *L. interrogans* were present in the blood circulation, a 50 µl drop of blood was collected every day post infection, and imaged *ex vivo*. Data showed that bioluminescent bacteria were indeed present in the blood (Fig. 1D). The light emitted was proportional to that measured in the whole mouse, showing exponentially growing *L. interrogans* in the blood between 1 and 3 dpi, which was confirmed by q-PCR data (Fig. 1D). Interestingly, although the bacteria used for the inoculation glowed equally well with or without ATP (Fig. 1D, lower panel), confirming that they were all alive at the time of inoculation, more light was emitted in the blood in the presence of ATP at 1 and 2 dpi, suggesting the presence of dying bacteria in the blood. No such difference in bioluminescence with or without ATP could be observed at 3 dpi. These findings suggest that the blood defenses were active at 1 and 2 dpi, and efficiently inactivated some bacteria, but were no longer efficient 3 dpi. Together, these results suggest that mice infected with 2×10^8^ *L. interrogans* succumbed because of the consequences of uncontrolled septicemia. Interestingly, the doubling time of leptospires in the blood calculated between 1 and 3 dpi was equivalent by live imaging (8.2±0.8 h n = 4) and qPCR (9.5±1.8h n = 4) and was shorter than culture in EMJH.

### Biphasic model of infection of mice with virulent bioluminescent *L. interrogans*

**Figure 2:**
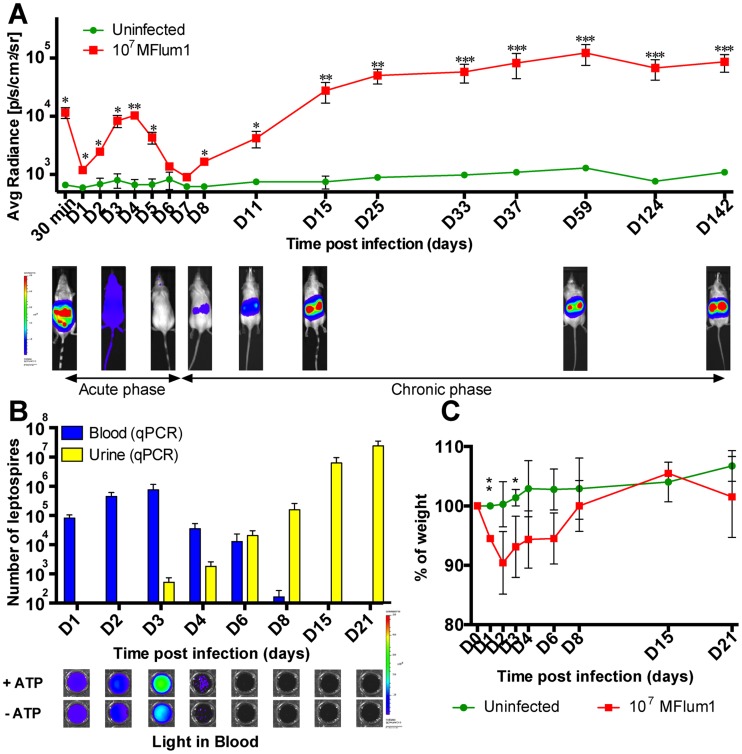
Kinetics of dissemination of bioluminescent MFlum1 in mice. All the bioluminescence analyses were performed after IP administration or addition of D-luciferin. (A) Live imaging tracking of 10^7^ MFlum1 IP injected to albino C57BL/6J mice. Images below the graph show the tracking of one infected mouse, photographed at different crucial time points. Data are expressed as the mean ± SEM of average radiance of light measured in photons/second/cm^2^ in n = 4 infected mice, imaged in the dorsal view, except for 30 min post-infection for which only imaging in the ventral view allows the visualization of the leptospiral dissemination in the peritoneal cavity. *p* values (*p<0.05, **p<0.01, ***p<0.001) between infected and uninfected groups. Images depict photographs overlaid with color representations of luminescence intensity, measured in photons/second/cm^2^ as indicated on the scales, where red is the most intense and purple the least intense. (B) Kinetics of leptospiral quantification by q-PCR in blood and urine of albino C57BL/6J mice infected with 10^7^ MFlum1. Below are shown corresponding images of the *ex vivo* live imaging of MFLum1 in blood, in the presence or absence of ATP. (C) Monitoring expressed as the percentage of weight loss of mice infected or not with 10^7^ MFlum1. Panels B and C are representative of 2 independent experiments with a total of n = 8 mice for each group. *p* values (*p<0.05, **p<0.01) between infected and uninfected groups.

The kinetics of leptospiral infection was studied in albino C57BL6/J mice after IP injection of 10^7^ MFlum1 bacteria *per* mouse, to avoid death of the mice (table 3). From 1 dpi and until 4 dpi, the bioluminescence increased, corresponding to the dissemination and rapid growth of leptospires in mice, then progressively decreased to disappear at 6 and 7 dpi, corresponding to an apparent clearance of the bacteria (Fig. 2A, lower panel). However, at 8 dpi, the bioluminescence reappeared, restricted to the kidneys and gradually increased for 1 or 2 weeks to reach a steady state (Fig. 2A). This renal chronic colonization was monitored for more than 14 months with no decrease of the bioluminescence (Fig. 2A and Fig. S2 in S1 Text). Similar observations were obtained with Balb/c mice, showing that the kinetics of leptospires dissemination and renal colonization were not unique to the C57BL6/J mice (Fig. S3 in S1 Text). Further characterization of the *L. interrogans*-infected albino C57BL6/J model was performed. First, the presence of leptospires in the urine and blood was monitored throughout the first weeks post infection (Fig. 2A). q-PCR data showed that bacteria were present in the blood of mice from 1 to 8 dpi, with a peak at 3 dpi, then a gradual decrease until 8 dpi, after which no further bacteria were detected (Fig. 2B). *Ex vivo* light imaging of the blood samples showed that live bacteria were present in the bloodstream until 3 dpi, but were not detected afterwards. Interestingly, at 2, 3 and 4 dpi, higher levels of light were measured from blood in the presence of ATP than in the absence, suggesting that dying bacteria, or released luciferase, were present in the blood, and that blood defenses were killing some leptospires at the infectious dose of 10^7^ MFlum1 (Fig. 2B, lower panel). The doubling time of leptospires in blood between 1 and 3 dpi was equivalent by live imaging (13.4±1.4 h) and q-PCR (11.8±1.3h), and was longer than the doubling time calculated for the lethal infection, suggesting better efficiency of the blood defense in this sub-lethal infection. The first 2 or 3 dpi were also characterized by weight loss of around 5 to 10%, followed by progressive recovery to the initial weight from 6 dpi to 8 dpi. From 15 dpi (Fig. 2C) to several months post infection (Fig. S4 in S1 Text), infected and uninfected control mice were identical in weights, suggesting an asymptomatic chronic infection. Consistent with the increasing bioluminescence observed in the kidneys from the second week of infection, leptospires were detected in increasing numbers in the urine from 3 dpi by q-PCR (Fig. 2B). Together, these data suggest that the course of experimental IP infection with a sublethal dose of *L. interrogans* is biphasic (Fig. 2A), beginning with a mild acute disease (acute phase) during the first week of infection, characterized by septicemia associated with transient weight loss, and subsequent clearance of the bacteria from the blood, then followed from the beginning of the second week by a chronic phase corresponding to the growth of *L. interrogans* in the kidneys, establishment of an asymptomatic renal colonization and excretion of leptospires in the urine.

### Threshold of leptospiral infective dose leading to renal colonization

**Figure 3:**
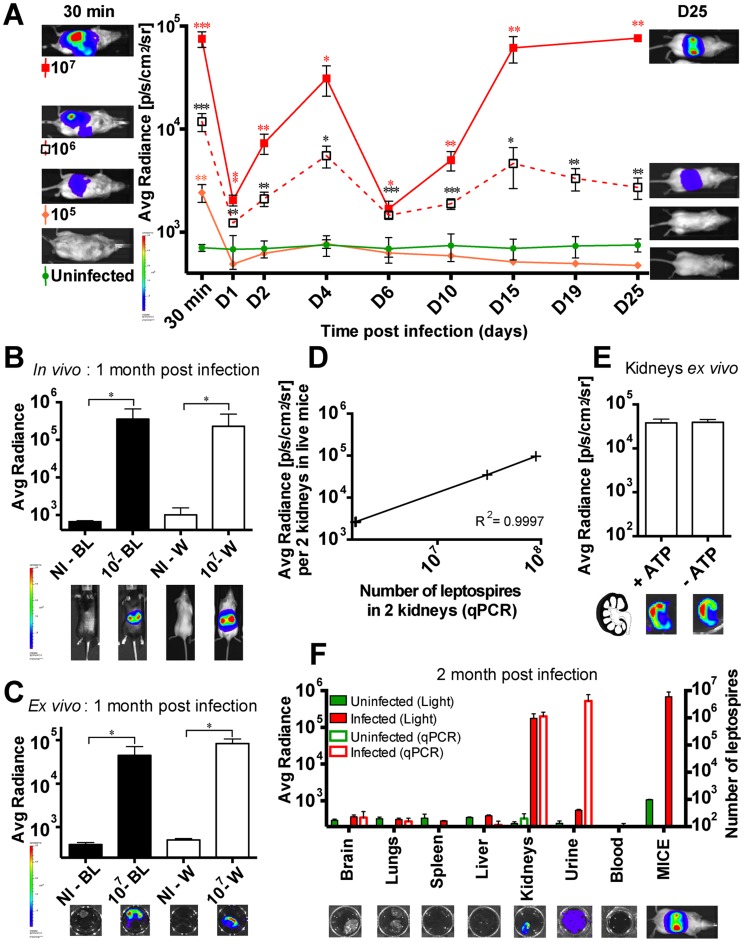
Threshold of infective dose of MFlum1 to obtain renal colonization. (A) Comparison of the dissemination kinetics for MFlum1 injected to albino C57BL/6J at different doses. Data are expressed as the mean ± SEM of average radiance of light measured in photons/second/cm^2^ in n = 4 infected mice, imaged in the dorsal view, except for 30 min post-infection for which only imaging in the ventral view allows the visualization of the leptospiral dissemination in the peritoneal cavity. On the right side are shown images of one representative mouse at 30 min post infection as a control of infection and on the left side, images of one representative mouse at the end of the kinetics. *p* values (*p<0.05, **p<0.01, ***p<0.001) are indicated in corresponding colors for each group *versus* the uninfected group. *In vivo* (B) and *ex vivo* (C) live imaging and quantification of albino (W) and black (BL) C57BL/6J mice one month post infection with (10^7^) or without (NI) 10^7^ MFlum1. Bioluminescence imaging in dorsal view was carried out after dorsal shaving of the black mice. Below are shown corresponding images of live mice gated on the kidneys (B) and *ex vivo* (C) tracking of half-kidneys after sacrifice and addition of D-luciferin. *p* value (*p<0.05) between infected and uninfected groups n = 4 mice *per* group. (D) Correlation between renal imaging and q-PCR. Bioluminescence imaging at 25 dpi in dorsal view of live chronically infected C57BL/6J mice (from 10^6^ or 10^7^ experiments), gated on the kidneys. Subsequently, mice were euthanized and kidneys were harvested and further processed for determination of the leptospiral load by q-PCR. Each cross represents an individual mouse. (E) *Ex vivo* imaging in presence or absence of ATP of half-kidneys from 10^7^ MFlum1 infected mice one month post infection. Data are expressed as the mean ± SEM of average radiance of light measured in photons/second/cm^2^ for 6 half-kidneys for each group. Below are shown corresponding images of the tracking of half-kidney after sacrifice and addition of D-luciferin. On the left is shown the schematic representation of an infected kidney in longitudinal cross section. (F) *Ex vivo* live imaging (Left Y axis) and the corresponding number of leptospires measured by q-PCR (Right Y axis) of different organs from 10^7^ MFlum1 infected mice, one month post infection. Data are expressed as the mean ± SEM of average radiance of light measured in photons/second/cm^2^. This panel represents 2 experiments with a total of n = 6 mice infected with MFlum1, and n = 5 naïve mice. Below are shown corresponding images of the live imaging of one representative organ or fluid and a mouse before the sacrifice. For imaging, urine was pooled from several mice, to obtain a minimum volume of 50 µL.

We next studied the kinetics of infection in albino C57BL/6J with different doses of MFlum1 from 10^5^ to 10^7^ bacteria *per* mouse. The kinetics of bioluminescence were similar using a dose of 10^6^ or 10^7^ leptospires *per* injection, and the kidneys of all mice were colonized (Fig. 3A). However, at a dose of 10^6^, the extent of the bioluminescence was reduced both at the acute phase of dissemination and at the chronic phase compared to the dose of 10^7^ (Fig. 3A). At the lower dose of 10^5^, no growth of bacteria or renal colonization occurred. These results suggest that the blood immune response is able to control a certain degree of *L. interrogans* infection, with efficient clearance of the bacteria in the first dpi, following a dose of 10^5^ or below. However, at higher infecting doses, although most of the bacteria were cleared at 6 dpi, *L. interrogans* succeeded in colonizing the kidneys. Hence, the threshold of the IP infection dose associated with renal colonization was 10^6^ bacteria *per* mouse, and interestingly, the extent of renal colonization was proportional to the infecting dose (Fig. 3A).

### Infected black C57BL/6J mice can be imaged at the chronic phase

In our previous studies of innate immune recognition of leptospires, we used transgenic mice in the C57BL/6J background. These mice have black fur, which prevents the measurement of emitted light. To investigate whether we could use the bioluminescent *Leptospira* strains in these mice, black and albino C57BL/6J mice were infected with 10^7^ MFlum1. One month post infection, the black mice were shaved dorsally before imaging. The levels of bioluminescence in live albino and in shaved black C57BL/6J mice were equivalent (Fig. 3B). To be sure that the infected organs were the kidneys, mice were sacrificed and kidneys removed, then transferred to a D-luciferin solution and imaged. Bioluminescence was indeed observed in the kidneys, and was equivalent in white and black mice (Fig. 3C). These results show that it is feasible to use black mice to monitor chronic renal leptospirosis using bioluminescent *L. interrogans*.

### Bioluminescence measured during the chronic phase is proportional to bacterial load

We next wanted to investigate whether the bioluminescence measured in live mice during the chronic phase in the kidneys reflected the bacterial load. At 25 dpi, MFlum1-infected mice were imaged gating on the kidneys, then mice were sacrificed and both kidneys used for quantification of *L. interrogans* by q-PCR (Fig. 3D). The results showed that the number of bacteria in kidneys measured by q-PCR was strictly proportional to the emitted light in live mice. However, the emitted light was surprisingly low compared to the number of leptospires inferred by q-PCR, suggesting either that a high number of leptospires were not bioluminescent or that light emission was impaired. *Ex vivo* imaging of longitudinally sectioned half-kidneys, in the presence or absence of ATP, showed no difference in light (Fig. 3E), suggesting that the leptospires present in the kidneys were healthy and not in the process of being degraded (Fig. 3E, lower panel). Moreover, imaging suggested a cortical localization of bacteria in the kidney (Fig. 3E, lower panel), consistent with the known localization of leptospires in the proximal tubules of kidneys.

### Kidneys are the reservoir of leptospires at the chronic phase

Because of the lower sensitivity of live imaging compared to q-PCR data in the kidneys, we wondered if, at the chronic phase, *L. interrogans* were restricted to kidneys as suggested by the light images of whole mice (Fig. 3B). MFlum1 infected mice were sacrificed 2 months post infection and their organs, blood and urine collected to analyze *ex vivo* the presence of leptospires. *Ex vivo* imaging of brain, lungs, liver, spleen and blood did not reveal any bioluminescence (Fig. 3F), suggesting a low amount or absence of leptospires in these organs. The lack of bacteria in these organs and blood was further confirmed by q-PCR data (Fig. 3F). By opposition, *L. interrogans* were detected in infected kidneys and urine, both by light imaging and q-PCR, as expected (Fig. 3F). Altogether, these data strongly suggest that kidneys constitute the only reservoir of leptospires at the chronic phase of infection in mice.

### Antibiotics administered early post-infection are efficient against *L. interrogans*

**Figure 4:**
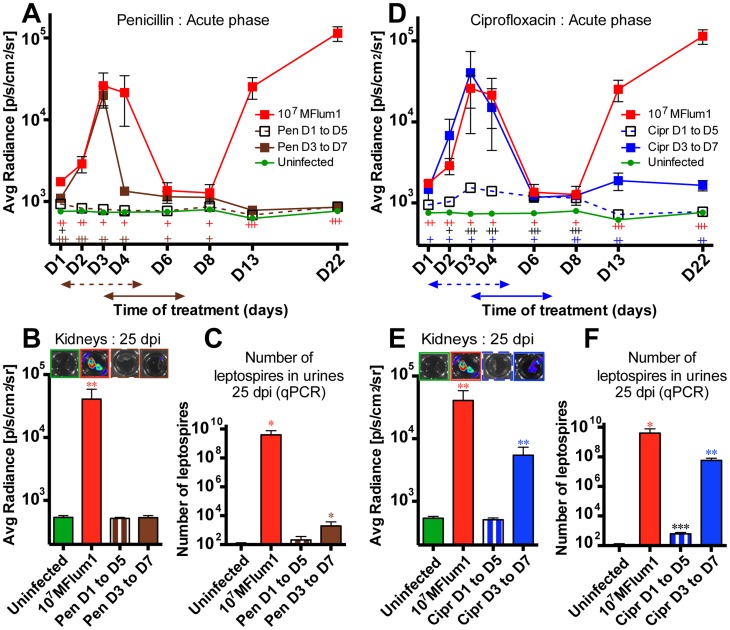
Effects of antibiotic treatments at the acute phase of leptospirosis. C57BL/6J mice were infected with 10^7^ MFlum1 (n = 4) and injected IP daily for 5 consecutive days with penicillin G (Pen) or ciprofloxacin (Cipr) from 1 until 5 dpi (D1 to D5) or from 3 until 7 dpi (D3 to D7). Thereafter, mice were sacrificed at 25 dpi. Comparison of the dissemination kinetics for 10^7^ MFlum1 injected in albino C57BL/6J mice, treated or not with penicillin G (A) or ciprofloxacin (D). Data are expressed as the mean ± SEM of average radiance of light measured in photons/second/cm^2^ in n = 4 for each group. *p* values (+*p*<0.05, ++*p*<0.01, +++*p*<0.001) are indicated in corresponding colors for each group *versus* the uninfected group. Panels (B) and (E) are corresponding bioluminescence images of half kidneys from mice sacrificed 25 dpi, from experiments depicted in panels (A) and (D), respectively. The arrows indicate the duration of the different treatments. Data are expressed as the mean ± SEM of average radiance of light measured in photons/second/cm^2^ gated on two half kidneys in n = 4 infected mice. *p* values (+*p*<0.05, ++*p*<0.01, +++ *p*<0.001) are indicated in corresponding colors for each group *versus* the uninfected group. Above are shown corresponding images of the *ex vivo* tracking of corresponding half-kidneys after sacrifice and addition of D-luciferin. Images depict photographs overlaid with color representations of luminescence intensity, measured in photons/second/cm^2^ and indicated on the scales, where red is most intense and purple is least intense. Panels (C) and (F) correspond to leptospiral load in urine at 25 dpi, measured by q-PCR, from experiments depicted in panel (A) and (D), respectively. Data are expressed as the mean number of leptospires in 100 µl of urine ± SEM for n = 4 infected or uninfected mice. *p* values (*p<0.05, **p<0.01, ***p<0.001) are indicated in corresponding colors for each group *versus* the uninfected group.

We next used the bioluminescent *L. interrogans* to test treatments able to combat leptospirosis. First, we focused on antibiotics administered during the acute phase of infection. Penicillin G was used because it is the treatment of choice for acute leptospirosis in humans, and was administered once a day through the intraperitoneal route for 5 consecutive days to mice already infected with 10^7^ MFlum1. Mice were treated with the antibiotic according to two protocols, either from 1 to 5 dpi, to target the dissemination phase, or from 3 until 7 dpi to let the bacteria disseminate before treatment, as performed previously [15]. Mice were imaged daily during the acute phase, and at different time points during the chronic phase. Mice were sacrificed at 25 dpi and the kidneys imaged and urine checked by q-PCR for the presence of leptospires. Administration of penicillin G from 1 dpi abrogated the bioluminescence in live infected mice, and no recurrence of light occurred later in the kidneys (Fig. 4A). This suggested that the bacteria had been cleared, which was confirmed as leptospires were not detected by q-PCR in the urine at 25 dpi (Fig. 4B). When administered from day 3 p.i., although the bioluminescence observed at the chronic phase was close to the background, some bioluminescence was still detected at 8 dpi. (Fig. 4A), suggesting live bacteria were present in the kidneys. Moreover, after dissection at 25 dpi, although no bioluminescence was detected in the kidneys, low levels of leptospires were detected in the urine by q-PCR (Fig. 4C). These results suggest that a few bacteria reached the kidneys before 3 dpi and escaped the D3–D5 penicillin treatment resulting in a sub detectable colonization of the kidney.

We wondered whether ciprofloxacin, a quinolone often used in urinary tract infections could be more active than penicillin to eradicate leptospires. Bioluminescence tracking showed that ciprofloxacin was active to avoid renal colonization by leptospires when administered from 1 to 5 dpi (Fig. 4D). However, the D1–D5 ciprofloxacin treatment was less active than the one with penicillin G, since some dissemination and growing of the bacteria still occurred at the acute phase (Fig. 4D), and despite undetectable bioluminescence in the kidneys at 25 dpi, some bacteria were detected in the urines. The ciprofloxacin administered from 3 dpi was not able to block the renal colonization (Fig. 4D). Imaging of dissected kidneys and urine screening for the presence of *L. interrogans* confirmed these results (Figs. 4E and 4F). Together, these findings showed the feasibility of using bioluminescent *L. interrogans* strains to monitor the effects of drugs against leptospirosis, and suggest that penicillin treatment, better than ciprofloxacin, is efficient to eradicate leptospires if administered in the very first days after infection.

### Antibiotics administered during the chronic stage are not efficient to eradicate leptospires

**Figure 5:**
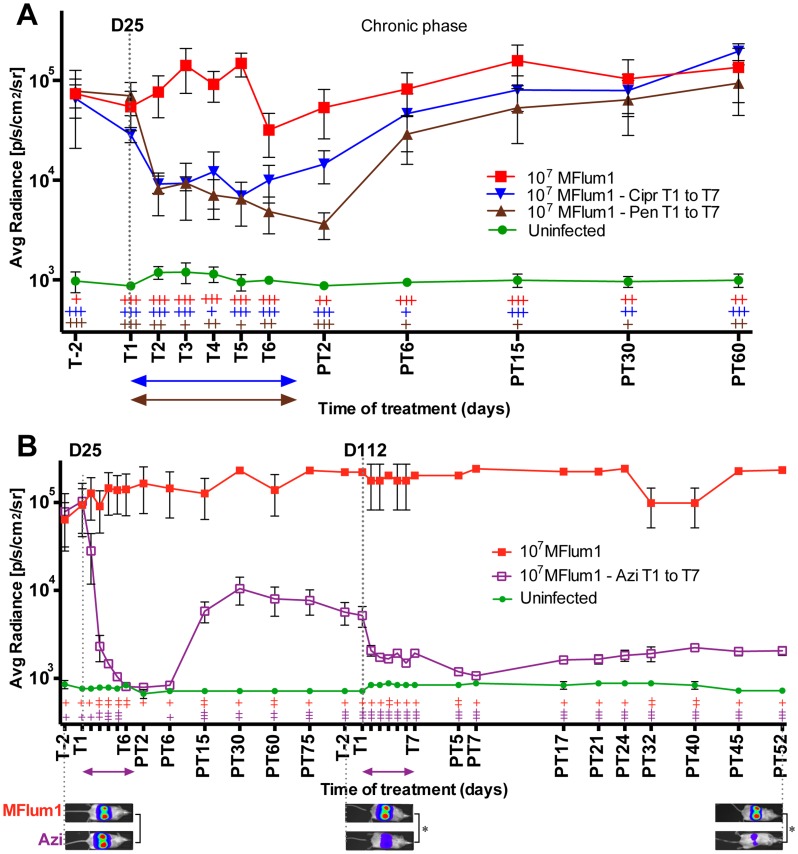
Effect of antibiotic treatments at the chronic phase of leptospirosis. C57BL/6J mice chronically infected for 25 days (D25) with 10^7^ MFlum1 were (A) IP injected daily for 7 days with penicillin G (Pen) (brown) or Ciprofloxacin (Cipr) (blue) from 25 until 31 dpi (T1 to T7), (B) injected daily for 7 days with azithromycin (Azi) from 25 until 31 dpi, and from 112 until 118 dpi (T1 to T7). The arrows indicate the duration of the different treatments. All the bioluminescence analyses were done after IP administration of D-luciferin. Data are expressed as the mean ± SEM of average radiance of light measured in photons/second/cm^2^ in n = 4 infected treated or untreated mice. *p* values (+*p*<0.05, ++*p*<0.01, +++*p*<0.001) are indicated in corresponding colors for each group *versus* the uninfected group. Below are shown images of the tracking of one untreated, infected (MFlum1) mouse and an infected treated (Azi) mouse photographed at different crucial time points. The corresponding *p* values (*p<0.05) were calculated between infected untreated group and treated group (Azi). Images depict photographs overlaid with color representations of luminescence intensity, measured in photons/second/cm^2^ and indicated on the scales, where red is most intense and purple is least intense. PT, days post treatment.

To better understand whether the antibiotics could kill leptospires once they are settled in the kidneys, we next monitored the effects of both penicillin G and ciprofloxacin treatments administered during the chronic phase of infection. Mice infected for 25 days with 10^7^ MFlum1, harboring the leptospires in the kidneys, were treated once a day for 7 consecutive days with the antibiotics. Interestingly, initial bioluminescence levels dropped rapidly, stabilizing on the second day of treatment at a steady state, corresponding to an approximately 10-fold reduction in bioluminescence compared to non-treated, infected control mice. Two days after the end of the treatment, bioluminescence began to recover in the kidneys, and was fully restored by 2 weeks post treatment (Fig. 5A). These results suggest that only a fraction of the *L. interrogans* population present in their niche, presumably the renal proximal tubule, was successfully targeted by the antibiotics, and that the remaining *L. interrogans* were protected and fully recolonized their niche once the treatment was terminated.

We next tested azithromycin, a macrolide antibiotic, very efficient in combating Gram-negative bacteria and *L. interrogans* [24], with a very good tissue distribution [25]. Mice chronically infected with 10^7^ MFlum1 for one month were treated for 7 consecutive days with azithromycin. The bioluminescence decreased rapidly to background levels after 6 days of treatment, and was still not detectable 2 days after the end of the treatment, confirming the better efficacy of azithromycin compared to penicillin and ciprofloxacin to target leptospires in the kidneys (Fig. 5B). However, 6 days post treatment, the bioluminescence reappeared in the kidneys, reaching a steady state one month post treatment although at a lower level than was observed prior to treatment (Fig. 5B). To determine whether leptospires that reappeared were persistent bacteria that escaped the antibiotic, or were bacteria resistant to azithromycin, we waited for 2 months then retreated the mice for 7 days with the same concentration of azithromycin. The bioluminescence dropped rapidly to a level slightly above background, and continued to decline reaching levels close but significantly different from the background one week after the end of the treatment. However, 2 weeks post treatment, the bioluminescence again reappeared in the kidneys, at a low steady level (Fig. 5B). This second treatment was active suggesting that the bacteria did not acquire resistance to the azithromycin. These results suggest that each treatment with azithromycin succeeded to sterilize the whole leptospiral population of a relatively important number of tubules. However, in a few tubules, only a portion of the population was eliminated, and persistent bacteria were able to survive the azithromycin treatment, and refill their niche once the treatment was over.

### A single injection of azithromycin protects against acute and chronic leptospirosis

**Figure 6:**
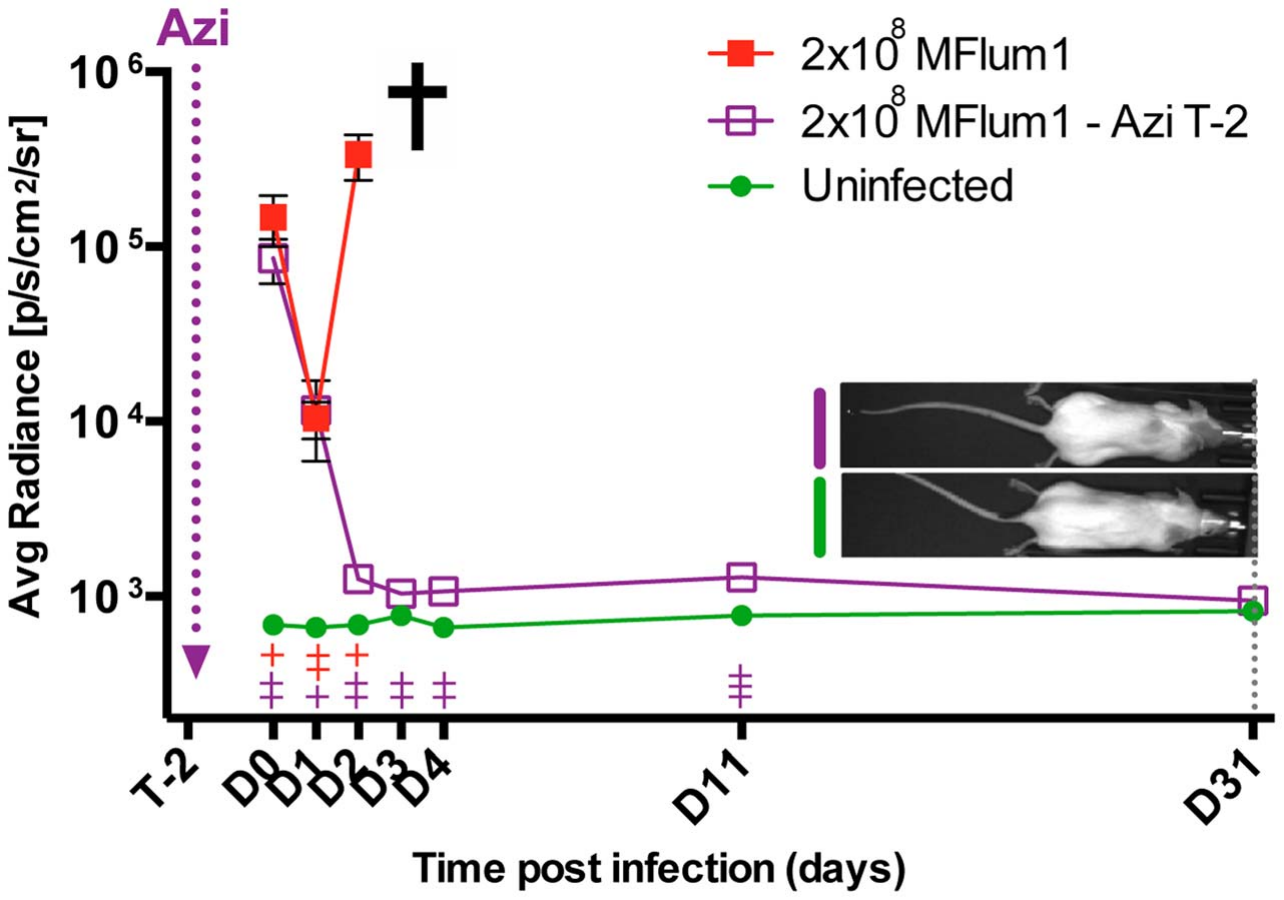
Azithromycin prophylactic treatment against leptospirosis. 8 C57BL/6J mice were infected with 2×10^8^ MFlum1 and 4 of them were injected IP two days before infection (T-2) with azithromycin (Azi). As controls, 4 uninfected mice were treated with azithromycin. All bioluminescence analyses were performed after IP administration of D-luciferin. Data are expressed as the mean ± SEM of average radiance of light measured in photons/second/cm^2^ in n = 4 mice imaged in the dorsal view, except for 30 min post-infection (D0) for which only imaging in the ventral view allows the visualization of the leptospiral dissemination in the peritoneal cavity. *p* values (+*p*<0.05, ++*p*<0.01, +++*p*<0.001) are indicated in corresponding colors for each group *versus* the uninfected treated group. The cross indicates that the mice died or were sacrificed because of acute leptospirosis. On the right side are shown images of the last tracking point (24 dpi) of one treated-infected mouse compared to an uninfected treated mouse.

Because azithromycin showed the best efficiency against *L. interrogans*, and has an extended half-life of more than 48 h [25], we wondered whether it could be used as a prophylactic treatment against leptospirosis. To test its efficiency against acute leptospirosis, naïve albino C57BL/6J mice were treated with a single dose of azithromycin 2 days before infection with a lethal dose of 2×10^8^ MFlum1. Data showed that from 1 dpi, the bioluminescence increased in the infected control mice, but decreased to background levels in the azithromycin treated mice. Control infected mice suffering from acute leptospirosis had to be sacrificed at 3 dpi whereas the azithromycin pre-treated mice did not show any clinical signs of acute leptospirosis (Fig. 6). The same pre-treatment was also administered to Myd88 ko mice infected with 2×10^8^ MFlum1. We previously showed that Myd88 ko mice are very sensitive to, and die from, acute leptospirosis because of a lack of TLR4 recognition [8]. The azithromycin pretreated Myd88 ko mice were protected from death induced by the lethal dose of MFlum1, showing that sensitive hosts can also be protected by prophylactic azithromycin treatment. Importantly, 3 months after the infection, bioluminescence was not observed in the kidneys of treated mice (Fig. S5 in S1 Text). As a control, urine of all infected mice was checked by q-PCR. Urines of mice pre-treated with azithromycin were free of leptospires showing that the azithromycin treatment efficiently counteracted the lethal dose of *L. interrogans* in both resistant and susceptible hosts. These findings suggest that administration of a single dose of azithromycin can be very useful as a prophylaxis treatment against acute leptospirosis that also protects against renal colonization by *L. interrogans*. In comparison, an equivalent prophylactic antibiotic treatment performed with doxycycline, at the relative dose used in prophylaxis against human leptospirosis [26], was not successful to protect mice from the *L. interrogans*-induced death and renal colonization, since 2 out of 4 mice died from acute leptospirosis, and the 2 surviving mice harbored renal colonization (Fig. S6 in S1 Text).

## Discussion

In this study, bioluminescent strains of pathogenic *L. interrogans* serovar Manilae were constructed and successfully used *in vivo* to determine and characterize the kinetics of dissemination of *L. interrogans* in mice. This new model proved to reproduce both acute and chronic leptospirosis and was further used to test the efficacy of antibiotics.

Pathogenic *Leptospira* strains are poorly transformable. Targeted mutagenesis in pathogenic leptospires is thus very scarce, and libraries of transposed mutants of *L. interrogans* are still incomplete [27]. In this study, transformation of *L. interrogans* serovar Manilae, with the transposon carrying the luciferase cassette driven by the strong promoter *flgB*p, resulted in several random mutants, the majority of which exhibited insertions in coding sequences. For example, we obtained a mutant in the gene encoding a collagenase, which was recently shown as a virulence factor [28]. Further analysis of these mutants, or of others obtained with the strong promoter (*groES*p) (Table S1 in S1 Text), may increase our understanding of virulence factors in *L. interrogans*, including *L. interrogans* serovar Copenhageni strain Fiocruz, for which we recently obtained bioluminescent transformants.

We used the intergenic transformant MFlum1 to study infection *in vivo*. MFlum1 retained its virulence, suggesting that the biological cost of expression of the luciferase cassette had only a minor impact on its fitness. The bioluminescence correlated with the number of leptospires in the exponential phase of growth. The limit of detection of live imaging was around 10^3^ bacteria/ml, corresponding to the detection of 100 MFlum1 in 100 µl. This level of detection was higher than the luminescence detection limit, previously found with a *luxCDABE* transformant (around 10^4^ leptospires) [13]. Compared to the luciferase expressed from the *luxCDABE* operon, the increased light emission of firefly luciferase in the presence of the exogenous D-luciferin substrate probably contributes to the ability of our bioluminescent strains to be imaged in live mice.

The technique of live-imaging detection of bioluminescent leptospires brings for the first time a dynamic view of the infectious process. Live imaging of luciferase activity was less sensitive compared to q-PCR, but the advantage is that only live, and therefore infectious, bacteria are detected, which is not the case with q-PCR. We were unable to explain why low levels of bioluminescence were observed in the kidneys or in urine, compared to the number of leptospires detected by q-PCR. Also, we could not detect leptospires by live imaging in the urine of mice during the first 2 weeks post infection, although they were alive in the kidney and found by q-PCR in urine. It seems unlikely that the leptospires were dead in urine and most probably several factors known to impact the luciferase activity may have contributed to quenching of light emission, such as isoflurane anesthesia, which inhibits luciferase activity [29], or the pH or oxygen levels [30], which may not be optimal or evenly distributed throughout the tubules or in urine.

In opposition to hamsters, gerbils and Guinea pigs which are accurate models for acute human leptospirosis, mice are considered relatively resistant to acute leptospirosis [4]. Indeed, in previous studies, no lethality or severe clinical symptoms were observed in C57BL/6 mice after intraperitoneal infections with *L. interrogans* serovar Copenhageni strain Fiocruz, even at the high infective dose of 2×10^8^ bacteria [8], [16]. However, we and others have shown that mice, immunosuppressed or genetically deficient for TLR4 or B cells, proved to be more sensitive, and develop lethal acute leptospirosis, demonstrating the usefulness of these murine models to unravel key components of the innate defense against leptospirosis [5], [8], [31]–[33]. Here, C57BL/6 mice infected with high doses of the bioluminescent *L. interrogans* serovar Manilae, suffered from major septicemia and died within a few days. This model of septicemia induced by a highly virulent *L. interrogans* is novel and may be very useful to understand the pathophysiological consequences of acute leptospirosis, which in humans has a high case/fatality rate [4], [34]. Moreover, we showed the feasibility of this model to test therapeutic strategies interfering with the growth of bacteria in blood, such as penicillin G treatment administered early after infection, which efficiently cleared the infection in mice. In this vein, an important result was the success of the prophylactic treatment that prevented, with only one dose of azithromycin, both acute and chronic leptospirosis in resistant and susceptible Myd88 ko mice, which like humans are susceptible to leptospirosis [8]. It is tempting to hypothesize that this prophylactic treatment could be transposed to humans to avoid potential leptospirosis in different situations, such as outbreaks or athletic events.

At sublethal infective doses, the live imaging revealed a biphasic disease with a self resolving septicemia within the first week, followed by renal colonization, and chronic shedding of leptospires in the urines, closely matching the leptospiremia and leptospiruria phases classically described leptospirosis [2]. Of note, we showed that during the chronic phase, kidneys constitute the only reservoir of leptospires, since no DNA from leptospires could be amplified by q-PCR from blood nor from other organs known to be the target of leptospires at the acute phase such as the brain, liver, lungs and spleen [2]. Moreover, live imaging allowed us to roughly map the presence of leptospires in the renal cortex of the kidney, which is in accordance with the reported localization of leptospires in the proximal tubules of the nephrons [35]–[37].

The innate blood defense consists in phagocytes, antimicrobial peptides, natural IgM and complement proteins, that together participate in the clearance of microbes. *L. interrogans* are known to escape the human complement system by several mechanisms [38], [39]. In mice, our study suggests that *L. interrogans* are well controlled by the blood defense, at an infectious dose of up to 10^5^ bacteria, since at this dose, we observed a fast decrease of live bacteria and no further growth of leptospires. For higher infectious doses of 10^6^ or 10^7^ bacteria, leptospires were able to exponentially grow in the blood in the first 3 dpi, showing that the blood defense was partially ineffective, presumably because it was overwhelmed. In agreement with this hypothesis, we showed that the blood defenses inactivated some leptospires in the first days following the infection. Indeed, *ex vivo* live imaging of blood the presence or absence of ATP, highlighted the presence of metabolically inactive bacteria still harboring or releasing active luciferase. Moreover, the doubling time of MFlum1 in blood was higher in the sub-lethal infection than in the lethal infection, showing that the blood defense more efficiently combated the 10^7^ leptospires than 2×10^8^. The peak of bacteremia in blood occurring 3 dpi and strikingly, the subsequent complete clearance of leptospires from the blood, is in accordance with our previous study, which showed that a specific IgM response against leptospiral LPS was already mounted 3 dpi, and was partially protective against leptospires [8].

**Figure 7:**
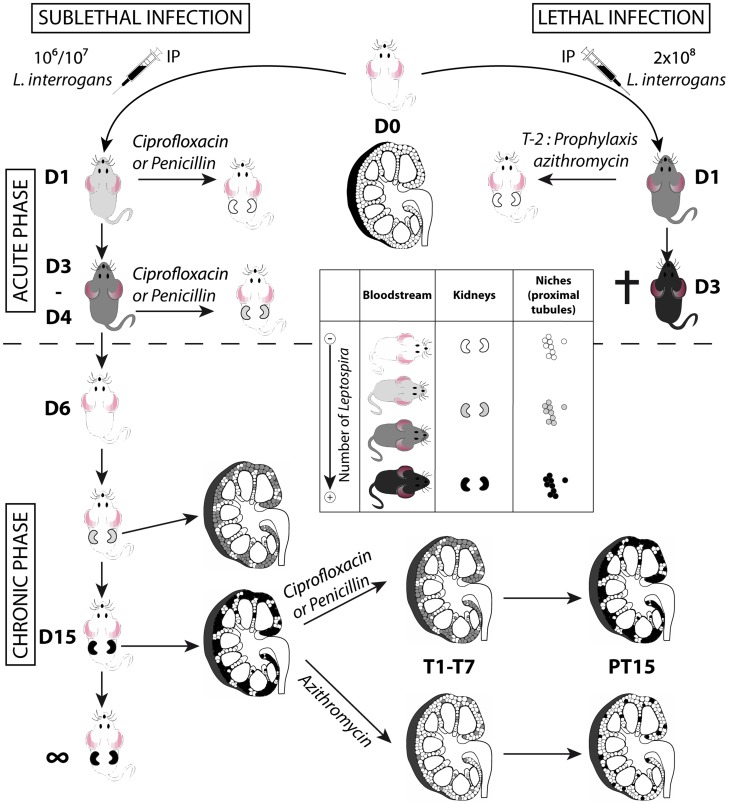
Model of acute and chronic leptospirosis in mice. This figure depicts the course of leptospirosis in mice following an IP infection with a lethal dose of bioluminescent MFlum1, leading to a septicemia or with a sub-lethal dose leading to a chronic leptospirosis, and the effects of different antibiotics administered at the acute (upper part of the figure) or chronic phase (lower part of the figure) of the leptospiral infection. Mice depicted without kidneys represent mice at the acute phase of infection. Mice depicted with kidneys represent mice at the chronic phase. Inside the kidneys schemed in longitudinal cross-section, the niches colonized by leptospires, presumably the proximal part of renal tubules (proximal tubules) are depicted by small circles. A grey color scale indicates the degree of leptospiral infection, where white means free of leptospires and black means a maximum of infection or colonization. The cross indicates that the mice died or were sacrificed because of acute leptospirosis. D1 to D15: days post-infection. T1-T7 duration of antibiotic treatments. PT15: 15 days post treatment. T-2 prophylaxis treatment 2 days prior to infection.

The fact that antibiotic treatments efficiently blocked renal colonization if administered 1 dpi, but partially failed if administered 3 dpi, together with the fact that leptospires were alive in the blood only during the first 3 dpi, suggests that the window of time for leptospires to escape the blood defense and reach their niche, is restricted to the exponential growth phase, between the first and third dpi. Moreover, the dose response and azithromycin-treatment experiments suggest that the extent of renal colonization by leptospires depends on the initial early access of a few leptospires to a restricted number of niches (Fig. 7). Indeed, a lower dose of infection reduced the burden of stable renal colonization. At the very beginning of the chronic phase, the regular increase of bioluminescence suggested that leptospires were protected from the immune response, which was highly effective since all leptospires disappeared from the circulation. Hence, within 2 to 3 weeks, the leptospires replicated to fill up their niche, reaching a steady level where the shedding and replication rates were in equilibrium. Since no increase in renal colonization was observed over time, even in heavily colonized mice, our results also suggest that there was no cross colonization of new nephrons. Together these results indirectly suggest that the potent IgG response, known to occur in natural or experimental leptospirosis [8], [40], efficiently contained the leptospires in their niche.

Antibiotic treatments with penicillin or ciprofloxacine were effective against *L. interrogans* if administered early after infection. However, administered at the chronic phase, both penicillin and ciprofloxacin failed to eradicate all the leptospires from the tubules. Therefore once the treatment was over, the leptospires regrew in the tubules and the levels of renal colonization 15 days post treatment were identical to those before treatment (Fig. 7). These results are in line with data from the literature, showing that penicillin or other antibiotics proved efficient in patients, only when given early after the onset of leptospirosis [41]. By contrast, azithromycin efficiently cleared the infection in most of the renal tubules, and leptospires persisted in only a few tubules that were subsequently fully recolonized (Fig. 7).

Doxycycline has been used in the past as a weekly chemoprophylaxis treatment in different situations of outbreaks [42], [43], but it did not efficiently eradicate leptospires in prophylaxis treatment in our mouse model of lethal infection. This result is in line with a recent study showing that prophylaxis with a single dose of 200 mg of doxycycline in humans may not be 100% effective to protect against development of acute leptospirosis [26]. Moreover, although doxycycline was previously shown to clear leptospires more efficiently than ampicillin and ofloxacin in hamsters when administered early post infection [44], it did not efficiently clear leptospires from the hamsters kidneys when administered 4 dpi [45]. Together, these data suggest that doxycycline would have been less effective than azithromycin for clearance of leptospires at the chronic phase in mice. However, our results have to be carefully considered since we did not take into account the pharmacokinetic-pharmacodynamic parameters of antibiotics that differ between mice and humans [46]. Indeed, both ciprofloxacin and doxycycline have reduced half-lives in serum of mice compared to humans. Moreover, the daily doses of antibiotics used in our study were in the high range of what is usually administered to patients [41], but mice were injected only once a day, whilst patients usually receive antibiotics twice a day. Therefore, it is still possible that a longer course of azithromycin or adequate doses of ciprofloxacin and doxycycline would have been more efficient in eradicating the leptospires.

Leptospires have been shown to establish biofilms *in vitro* [47]. In *L. interrogans* infected mice, we observed at the chronic phase, strong labeling of the lumen of tubules with LipL32 antibodies [15], close to the dense colonization of renal tubules by leptospires previously observed in experimentally infected rats [48]. Biofilms are known to confer bacterial resistance to antibiotics [49] and our hypothesis is that the localized resistance of leptospires to antibiotics would be linked to biofilm formation in the proximal renal tubules of mice. Further studies aiming to test new therapeutic treatments, combining antibiotics and drugs to disperse biofilms, should be envisioned to potentiate the renal elimination of leptospires.

Chronic renal carriage of leptospires has recently been shown in mammals other than rodents, such as sea lions [50], cats [51], dogs [52] and most strikingly, in humans [53], [54]. The dogma that some rodents, such as rats, are a chronic carrier of the disease, whereas other animals are accidental hosts, may be simplistic, and asymptomatic carriage in humans may have been largely overlooked. Our results should alert about the potential incomplete elimination of leptospires after antibiotherapy, and the risk of chronic renal carriage of leptospires in both veterinary and human medicine. A recent study showed that most patients that have been hospitalized for acute leptospirosis still exhibited diverse symptoms associated with leptospirosis such as myalgia and headaches, 2 years after treatment [55]. These patients should be investigated through urine testing for potential chronic renal carriage of leptospires. In Taiwan, the incidence of chronic kidney disease is higher than in other parts of Asia, and may be linked to asymptomatic leptospirosis, since almost 10% of hospitalized patients with chronic kidney disease were found seropositive for leptospires, without reported history of the disease [56]. It would also be instructive to test these seropositive patients for urinary shedding of leptospires.

We studied here only the kinetics of infection upon intra-peritoneal injection of leptospires, which may reflect part of the infectious process, but not the initial events of leptospires penetrating the mucosa or the abraded skin. Further study is ongoing to better understand the consequences of more physiological routes of infection with pathogenic *L. interrogans*, without experimental breaches of the skin, such as by depositing bioluminescent *L. interrogans* on the eye, nose, mouth or skin. The bioluminescent strains will also be useful to study leptospirosis by live imaging in the young guinea pig, which constitutes a good animal model for acute leptospirosis, presenting pulmonary hemorrhages [6], or in the rat, the choice model of chronic leptospirosis [57]. Indeed, both Lewis rats and Hartley guinea pigs are albino and small enough to fit in the IVIS Spectrum machine.

In conclusion, this study, together with our previous work revealing mild fibrosis in kidneys from mice chronically infected with *L. interrogans* [15], definitively demonstrates that the mouse is a good experimental model to study leptospirosis. This work also provides the proof of principle that bioluminescent *Leptospira* strains will be useful tools to challenge vaccines, or to test therapeutic treatments. The study of survival, persistence and transmission of leptospires between the environment and rodents can also be envisioned.

## Supporting Information

Text S1 **Supporting information.** Table S1. List of bioluminescent *groES*p-luciferase transformants of *L. interrogans* serovar Manilae. Figure S1. Kinetics of bioluminescent *L. biflexa* serovar Patoc in mice. Figure S2. Renal colonization is stable over time. Figure S3. Kinetics of dissemination of bioluminescent MFlum1 in Balb/c mice. Figure S4. Chronic renal colonization with *L. interrogans* does not result in weight loss. Figure S5. Controls 3 months post infection of absence of renal colonization in mice prophylactically treated with azithromycin. Figure S6. Doxycycline prophylaxis experiment.
(PDF)
