# Supplementary material for: Live Imaging of Bioluminescent Leptospira interrogans in Mice Reveals Renal Colonization as a Stealth Escape from the Blood Defenses and Antibiotics
Source: PLoS Negl Trop Dis. 2014 Dec 4;8(12):e3359. doi: 10.1371/journal.pntd.0003359 (PMC4256284; doi:10.1371/journal.pntd.0003359)
Supplement: Text S1 — Supporting information. Table S1. List of bioluminescent groESp-luciferase transformants of L. interrogans serovar Manilae. Figure S1. Kinetics of bioluminescent L. biflexa serovar Patoc in mice. Figure S2. Renal colonization is stable over time. Figure S3. Kinetics of dissemination of bioluminescent MFlum1 in Balb/c mice. Figure S4. Chronic renal colonization with L. interrogans does not result in weight loss. Figure S5. Controls 3 months post infection of absence of renal colonization in mice prophylactically treated with azithromycin. Figure S6. Doxycycline prophylaxis experiment. (PDF) [file pntd.0003359.s001.pdf]

Text S1

Table S1. List of bioluminescent *groES*p-luciferase transformants of *L. interrogans* serovar Manilae

Mutant name	Location of insertion [a]	Predicted function of mutated gene or description
MGlum1	*LMANv1_8410002*<br>*LMANv1_8420001*	*Putative diguanylate phosphodiesterase – Hypothetical protein*
MGlum2	LMANv1_310018	ATPase and permease components of ABC-type multidrug transport system
MGlum3	LMANv1_4270005	Putative hydrolase
MGlum4	*LMANv1_3480001*<br>*LMANv1_3480002*	*Two putative lipoproteins*
MGlum5	LMANv1_4370006	Conserved protein of unknown function
MGlum6	LMANv1_7320006	Serine phosphatase RsbU
MGlum7	LMANv1_8200001	Putative lipoprotein
MGlum8	LMANv1_8420007	Protein of unknown function
MGlum9	LMANv1_90006	Conserved protein of unknown function
MGlum10	LMANv1_6580005	Serine/threonine phosphatase containing GAF domains
MGlum11	LMANv1_9140013	Putative potassium efflux transporter
MGlum12	LMANv1_7910004	Penicillin-binding protein 1 (PBP-1a)
MGlum13	*LMANv1_810009*<br>*LMANv1_830001*	*Two proteins of unknown function*
MGlum14	LMANv1_4100010	Conserved protein of unknown function
MGlum15	LMANv1_6460002	Potassium-transporting ATPase A chain
MGlum16	LMANv1_9780012	Putative acyltransferase
MGlum17	LMANv1_530008	Conserved protein of unknown function
MGlum18	*LMANv1_7610002*<br>*LMANv1_7610003*	*Sphingomyelinase C – Protein of unknown function*
MGlum19	LMANv1_tRNA21	End of Asn tRNA
MGlum20	*LMANv1_3950006*<br>*LMANv1_3950007*	*Conserved membrane protein – GTP-binding protein LepA*
MGlum21	LMANv1_4900001	Conserved exported protein of unknown function
MGlum22	LMANv1_1100003	LipL41
MGlum23	LMANv1_8450001	Fic protein involved in cell division
MGlum24	LMANv1_4380015	Anti-sigma factor antagonist
MGlum25	LMANv1_4490003	Conserved hypothetical protein; putative signal peptide
MGlum26	LMANv1_4160004	Conserved exported protein of unknown function
MGlum27	*LMANv1_2120012*<br>*LMANv1_2120013*	*Adenylate/guanylate cyclase – Conserved protein*
MGlum28	LMANv1_4790001	Conserved protein of unknown function
MGlum29	*LMANv1_1930003*<br>*LMANv1_1930004*	*Putative lipoprotein – Conserved protein*
MGlum30	*LMANv1_4160005*<br>*LMANv1_4160004*	*Putative arginyl-tRNA-protein transferase – Conserved exported protein*
MGlum31	*LMANv1_450004*<br>*LMANv1_450005*	*Putative lipoprotein – Conserved protein of unknown function*
MGlum32	LMANv1_7590002	Conserved membrane protein of unknown function

[a] as annotated in MicroScope (http://www.genoscope.cns.fr/agc/microscope/home/index.php)

italics: insertion in an intergenic region

The promoter *groE*p was amplified from *L. interrogans* Fiocruz L1-130 DNA (location chromosome 1 1648924-1649178) using the specific primers (pGroSF: TGTGCTAGCATGATTTGCAGTAGTTCCC and pGroSR: ATGCCATGGACTGACTCCTTAAAATTTATAAG) and was subcloned into pBAD::Luc with the NheI enzyme, resulting in the plasmid pGroES::Luc. The luciferase cassette was recovered from the pGroES::Luc plasmid using the NheI/ScaI enzymes. Plasmid pCj::GroE was generated by cloning the blunt-ended luciferase cassette into the blunt-ended PacI restriction site of the suicide conjugative plasmid pCjTKS2. Bioluminescent transformants of *L. interrogans* serovar Manilae (MGlum) were selected after conjugation with *E. coli* β2163 carrying pCj::pGroES.

Figure S1

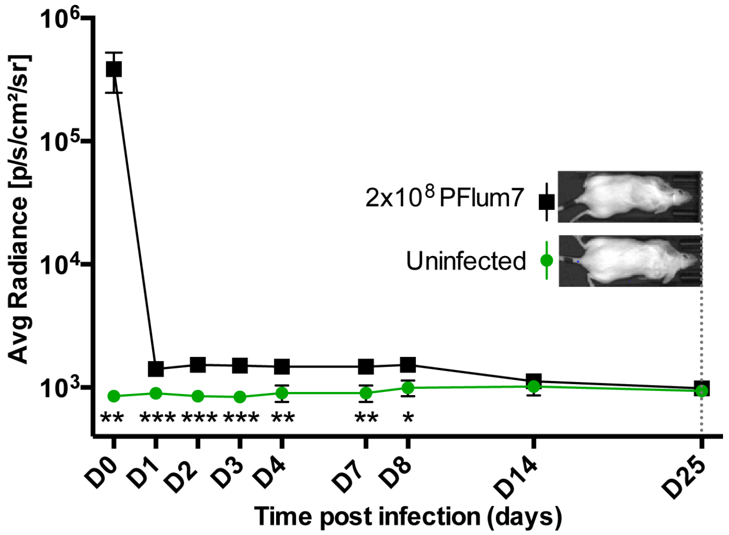

Fig S1. Kinetics of bioluminescent *L. biflexa* serovar Patoc in mice

Live imaging tracking over time of 2x10^8 bioluminescent *L. biflexa* Patoc PFlum7 injected intra-peritoneally (IP) into albino C57BL/6J mice. Bioluminescence analyses were carried out after the IP administration of D-luciferin. Data are expressed as the mean ± SEM of average radiance of light measured in photons/second/cm^2 in n=4 mice and imaged in the ventral view. *p* values ($*p < 0.05$, $**p < 0.01$, $***p < 0.001$) between infected and uninfected group. On the right side are shown images of the last tracking point (25 dpi) of one infected mouse compared to one uninfected mouse.

Figure S2

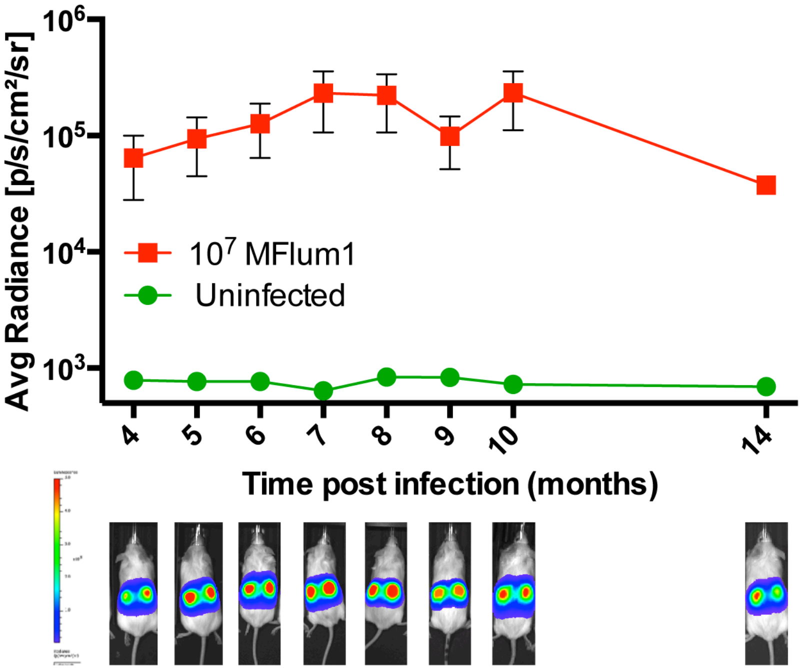

Fig S2. Renal colonization is stable over time

Live imaging tracking of 10^7 MFlum1 IP injected to albino C57BL/6J mice, between 4 and 14 months. Bioluminescence analyses were performed after IP administration or addition of D-luciferin. Data are expressed as the mean ± SEM of average radiance of light measured in photons/second/cm^2 in n=4 infected mice, imaged in the dorsal view. Images below the graph show the tracking of one infected mouse. Images depict photographs overlaid with color representations of luminescence intensity, measured in photons/second/cm^2 as indicated on the scale, where red is the most intense and purple the least intense. For infected mice, there were no statistically significant differences between means of groups, corresponding to imaging at the different time points, as determined by one-way ANOVA (p : 0,63).

Figure S3

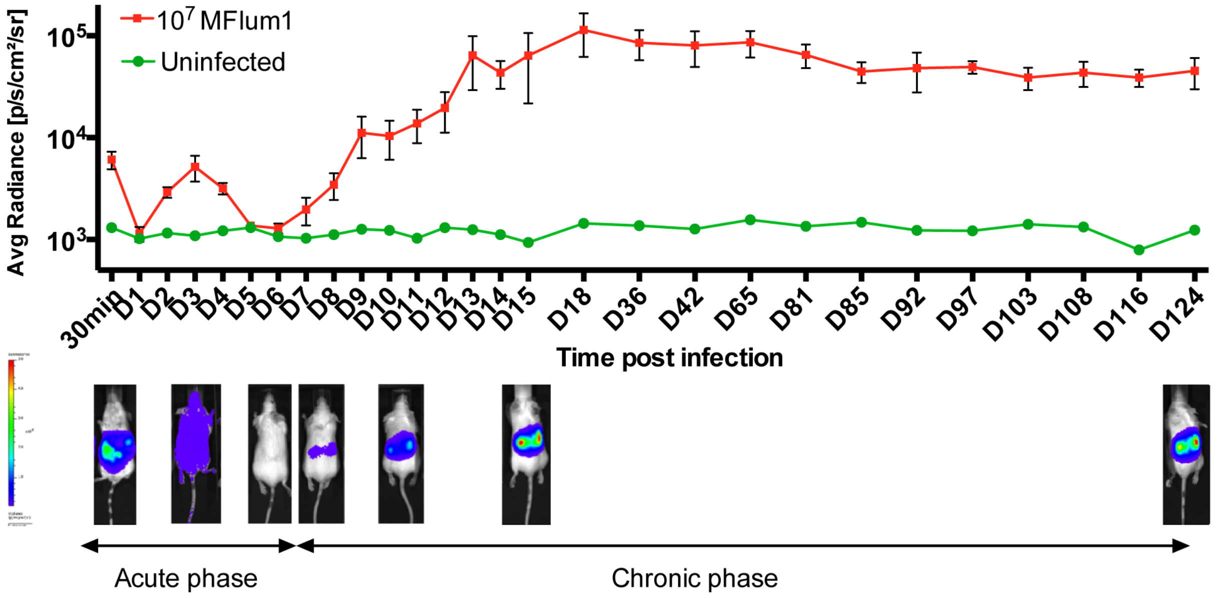

Fig S3. Kinetics of dissemination of bioluminescent MFlum1 in Balb/c mice

Live imaging tracking of 10^7 MFlum1 IP injected to Balb/c mice. Images below the graph show the tracking of one infected mouse, photographed at different crucial time points. All the bioluminescence analyses were performed after IP administration or addition of D-luciferin. Data are expressed as the mean ± SEM of average radiance of light measured in photons/second/cm^2 in n=4 infected mice, imaged in the dorsal view, except for 30 min post-infection for which only imaging in the ventral view allows the visualization of the leptospiral dissemination in the peritoneal cavity. Images depict photographs overlaid with color representations of luminescence intensity, measured in photons/second/cm^2 as indicated on the scale, where red is the most intense and purple the least intense.

Figure S4

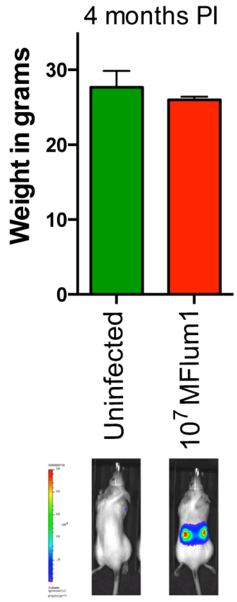

Fig S4. Chronic renal colonization with *L. interrogans* does not result in weight loss

Comparison of weight in grams between n=4 uninfected mice and n=4 infected mice with 10^7 MFlum1 4 months post-infection (PI). Images below the graph show the corresponding bioluminescence of an uninfected and an infected mouse. Bioluminescence analysis was performed after IP administration or addition of D-luciferin. Images depict photographs overlaid with color representations of luminescence intensity, measured in photons/second/cm^2 as indicated on the scales, where red is the most intense and purple the least intense.

Figure S5

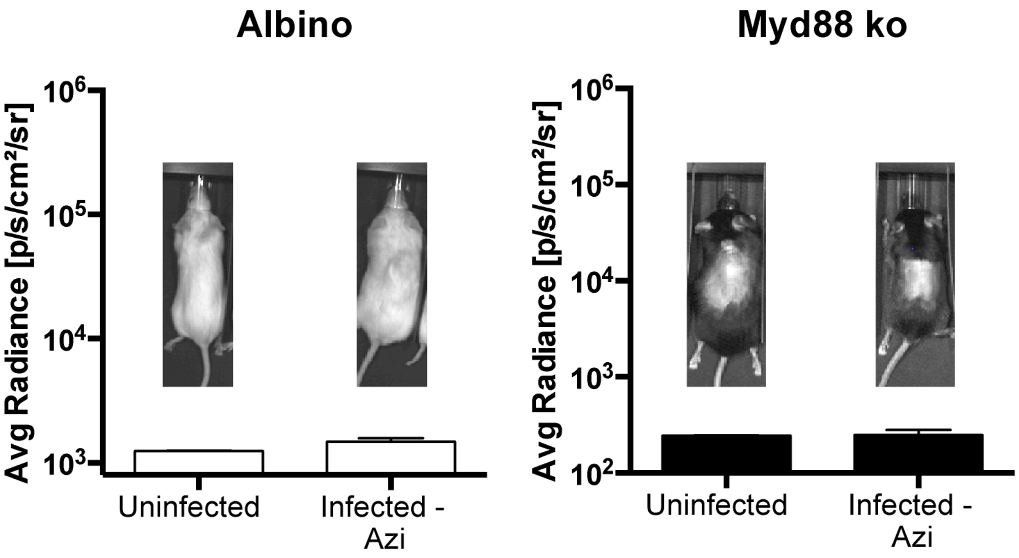

Fig S5. Controls 3 months post infection of absence of renal colonization in mice prophylactically treated with azithromycin

In vivo live imaging and quantification of albino and black Myd88 ko C57BL/6J mice 3 months post infection with (Infected-Azi) or without (Uninfected) 2x10^8 MFlum1. Infected-azi mice were injected IP two days before infection (T-2) with azithromycin. As controls, uninfected mice were treated with azithromycin, and the infected control mice died between 2 and 3 dpi for both albino and Myd88 ko C57BL/6J. Data are expressed as the mean ± SEM of average radiance of light measured in photons/second/cm^2 in n=4 mice imaged in the dorsal view, where imaging was carried out after dorsal shaving of the black mice. Above are shown corresponding images of live mice after IP addition of D-luciferin.

Figure S6

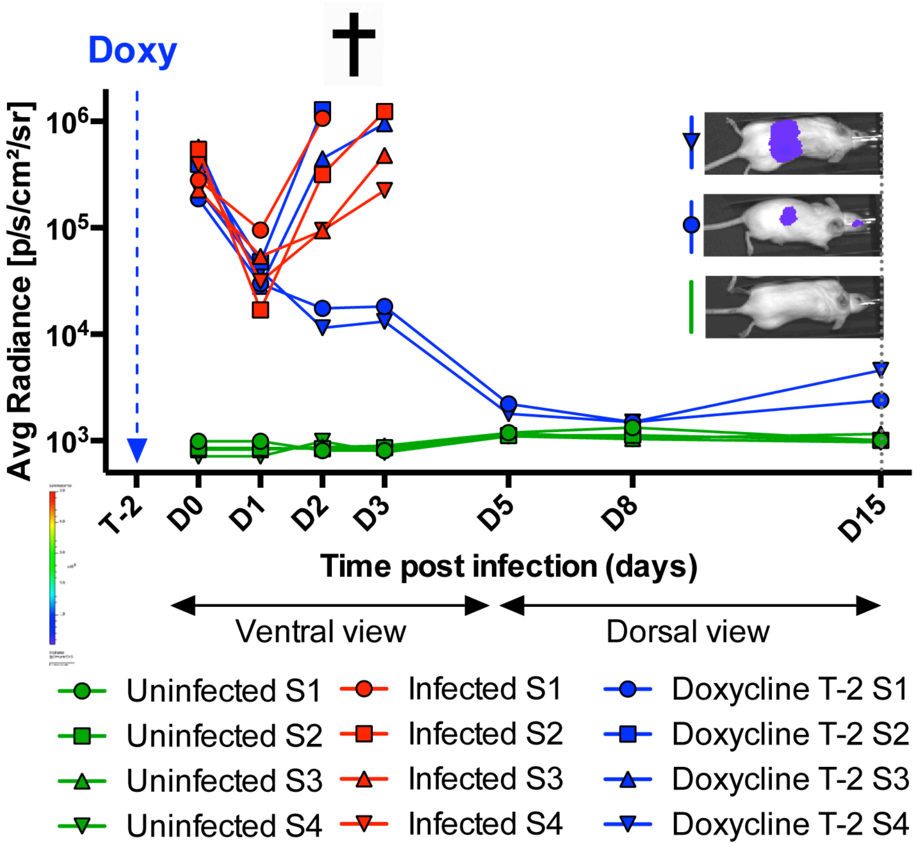

Fig S6. Doxycycline prophylaxis experiment

8 C57BL/6J mice were infected with $2x10^8$ MFlum1 and 4 of them were injected IP two days before infection (T-2) with a single dose of doxycycline (Doxy) (70µg/mouse which is equivalent to the human single dose of 200mg/60kg). As controls, 4 uninfected mice were treated with doxycycline. All bioluminescence analyses were performed after IP administration of D-luciferin. Data are expressed in average radiance of light measured in photons/second/cm^2 in every single mouse imaged in the ventral view between D0 and D5 for the visualization of the leptospiral dissemination and imaged in the dorsal view at D8 and D15 for the visualization of kidneys colonization. The cross indicates that the mice died or were sacrificed because of acute leptospirosis. On the right side are shown images of the last tracking point (15 dpi) of the 2 surviving treated-infected mice compared to a representative uninfected treated mouse.
